# Systemic lupus erythematosus dysregulates the expression of long noncoding RNAs in placentas

**DOI:** 10.1186/s13075-022-02825-7

**Published:** 2022-06-14

**Authors:** Hui-hui Li, Lin-tao Sai, Yuan Liu, Colman I. Freel, Kai Wang, Chi Zhou, Jing Zheng, Qiang Shu, Ying-jie Zhao

**Affiliations:** 1 Department of Obstetrics and Gynecology, Qilu Hospital, Cheeloo College of Medicine, Shandong University, Jinan, 250012 Shandong China; 2 Department of Obstetrics and Gynecology, University of Wisconsin-Madison, Madison, WI 53715 USA; 3 Department of Infectious Diseases, Qilu Hospital, Cheeloo College of Medicine, Shandong University, Jinan, 250012 Shandong China; 4 Clinical and Translational Research Center, Shanghai First Maternity and Infant Hospital, Tongji University School of Medicine, Shanghai, China; 5 School of Animal and Comparative Biomedical Sciences, University of Arizona, Tucson, AZ 85719 USA; 6 Department of Rheumatology, Qilu Hospital, Cheeloo College of Medicine, Shandong University, Jinan, 250012 Shandong China; 7 Shandong Provincial Clinical Research Center for Immune Diseases and Gout, Jinan, 250012 Shandong China; 8 Scholars Program, University of Nebraska Medical Center, Omaha, NE 68198 USA

**Keywords:** lncRNA, RNA sequencing, Systemic lupus erythematosus, Placenta

## Abstract

**Background:**

Systemic lupus erythematosus (SLE) can cause placental dysfunctions, which may result in pregnancy complications. Long noncoding RNAs (lncRNAs) are actively involved in the regulation of immune responses during pregnancy. The present study aimed to determine the lncRNA expression profiles in placentas from women with SLE to gain new insights into the underlying molecular mechanisms in SLE pregnancies.

**Methods:**

RNA sequencing (RNA-seq) analysis was performed to identify SLE-dysregulated lncRNAs and mRNAs in placentas from women with SLE and normal full-term (NT) pregnancies. Bioinformatics analysis was conducted to predict the biological functions of these SLE-dysregulated lncRNAs and mRNAs.

**Results:**

RNA-seq analysis identified 52 dysregulated lncRNAs in SLE placentas, including 37 that were upregulated and 15 downregulated. Additional 130 SLE-dysregulated mRNAs were discovered, including 122 upregulated and 8 downregulated. Bioinformatics analysis revealed that SLE-dysregulated genes were associated with biological functions and gene networks, such as regulation of type I interferon-mediated signaling pathway, response to hypoxia, regulation of MAPK (mitogen-activated protein kinase) cascade, response to steroid hormone, complement and coagulation cascades, and Th1 and Th2 cell differentiation.

**Conclusions:**

This is the first report of the lncRNA profiles in placentas from SLE pregnancies. These results suggest that the aberrant expression and the potential regulatory function of lncRNAs in placentas may play comprehensive roles in the pathogenesis of SLE pregnancies. SLE-dysregulated lncRNAs may potentially serve as biomarkers for SLE.

**Supplementary Information:**

The online version contains supplementary material available at 10.1186/s13075-022-02825-7.

## Background

Systemic lupus erythematosus (SLE) is a systemic autoimmune disease that predominantly affects women of reproductive age [1, 2]. During pregnancy, the loss of immune tolerance to the fetus in SLE may cause numerous maternal and fetal complications, including lupus flare, hypertension, preeclampsia (PE), eclampsia, spontaneous abortion, stillbirth, fetal growth restriction (FGR), neonatal lupus, and neonatal deaths [3–6]. The pregnancy outcomes in mothers with SLE and the well beings of fetuses born to SLE mothers have been improved tremendously over the last five decades due to the development of disease management [7, 8]. However, the mechanism underlying SLE-induced pregnancy complications remains unclear. Therefore, optimal disease control and multidisciplinary obstetrical care throughout gestation are essential to improve pregnancy outcomes in SLE.

SLE can cause placental dysfunction and insufficiencies such as decreased placental weight, ischemic hypoxic change, decidual vasculopathy and thrombi, fetal thrombi, and chronic villitis of implied unknown etiology during pregnancy [5]. These impaired placental functions may lead to numerous maternal and fetal complications, as mentioned above [9, 10].

Long noncoding RNAs (lncRNAs) are transcripts longer than 200 nucleotides in length without recognizable protein-coding potential [11]. LncRNAs play an essential role in regulating gene expression through multiple mechanisms [11–13]. LncRNAs also actively regulate key biological processes, including immune cell differentiation and immune responses [14]. Meanwhile, lncRNAs have been related to autoimmune diseases such as SLE, Sjögren’s syndrome, and rheumatoid arthritis [15–22].

LncRNAs are involved in the initiation and development of SLE via different signaling pathways, e.g., the nuclear factor-κB (NF-κB) signaling pathway [19], mitogen-activated protein kinase (MAPK) signaling pathway [20], and tumor necrosis factor (TNF) signaling pathway [16]. LncRNAs are also associated with SLE disease activity [20–22]. However, little is known about lncRNA expression profiles and functions in placentas from SLE pregnancy.

This study aims to investigate the SLE-dysregulated lncRNA expression profiles in placentas. Identifying SLE-dysregulated lncRNAs in placentas may aid us in identifying novel therapeutic targets and disease biomarkers for SLE. RNA sequencing (RNA-seq) was performed on placentas from SLE and normal full-term (NT) pregnancies. Bioinformatics analysis was conducted to reveal the underlying biological functions of dysregulated lncRNAs.

## Methods

### Subjects

All procedures were conducted in accordance with the Declaration of Helsinki. Tissue collection protocols were approved by the Institutional Review Board of Qilu Hospital, Shandong University and the Scientific and Ethical Committee of Shanghai First Maternity and Infant Hospital affiliated with Tongji University. Pregnant women with SLE (*n* = 10, with five female and five male fetuses) and NT (*n* =10, with five female and five male fetuses) were recruited from Qilu Hospital, Shandong University, and Shanghai First Maternity and Infant Hospital affiliated with Tongji University, respectively. Individuals excluded from the study include smokers and patients with cancer or diabetes mellitus. SLE was defined according to the American College of Rheumatology classification criteria [23]. The SLE disease activity index (SLEDAI) [24] was used to evaluate the disease activity of SLE patients. The questionnaire survey was used to collect demographic data and clinical manifestations of all patients. Laboratory data were obtained from the medical record. Clinical manifestations and laboratory records of all subjects are shown in Table 1 and Additional file 1: Table S1.

**Table 1:** Clinical and laboratory characteristics of the patients in the study

Characteristics	SLE (*n* = 10)	NT (*n* = 10)	*P*
Age (years), median (range)	29.0 (26–36)	30.5 (28–33)	> 0.05
BMI, median (range)	25.4 (23.0–32.3)	28.0 (21.8–32.7)	> 0.05
Gestation age (weeks), median (range)	38.5 (34.9–39.7)	39.1 (38.6–40.1)	< 0.05
Fetal weight (grams), median (range)	2950.0 (2150.0–3850.0)	3402.0 (2895.0–3730.0)	< 0.05
Fetal weight lower than 10th percentile (%)	10	0	> 0.05
Vaginal delivery (*n*)	8	8	> 0.05
Disease duration (months), median (range)	40.0 (10–167)	–	–
SLEDAI score, median (range)	2.5 (0–6)	–	–
ANA > 1:320, yes/no (*n*)	10/0	–	–
Anti-dsDNA, yes/no (*n*)	2/8	–	–
Anti-phospholipid, yes/no (*n*)	3/7	–	–
Preeclampsia, yes/no (*n*)	0/8	–	–
Proteinuria, yes/no (*n*)	3/7	–	–
Hypocomplementemia, yes/no (*n*)	3/7	–	–
Steroids, yes/no (*n*)	10/0	–	–
Immunosuppressive drugs, yes/no (*n*)	0/10	–	–
Aspirin, yes/no (*n*)	3/7	–	–

*SLE* systemic lupus erythematosus, *NT* normal term, *BMI* body mass index, *SLEDAI* systemic lupus erythematosus disease activity index, *ANA* antinuclear antibody

### Sampling and RNA isolation

Placentas were collected immediately after C-section or vaginal delivery. Six random biopsies from the fetal side of each placenta were sampled (using a 1 × 1 cm grid), snap-frozen in liquid nitrogen, and stored at −80 °C for further experiments.

Total RNA was isolated using the RNeasy mini kit (Qiagen, Germany). RNA concentration and quality were determined by the Qubit ® 2.0 Fluorometer (Life Technologies, USA) and the Nanodrop One spectrophotometer (Thermo Fisher Scientific Inc., USA). The integrity of total RNA was assessed using the Agilent 2100 Bioanalyzer (Agilent Technologies Inc., USA), and samples with RNA integrity number (RIN) values above 7.0 were used for sequencing.

### RNA-seq and data analysis for gene expression

RNA-seq analysis was performed on RNA samples from SLE and NT placentas (*n* = 8/group with three female and five male fetuses; Additional file 2: Table S2) using the VAHTS Total RNA-seq (H/M/R) Library Prep Kit (Vazyme, China) and Illumina NovaSeq 6000 platform (Illumina, USA) as described in Methods in Data Supplement. Differential expression gene (DEG) analysis for lncRNA/mRNA was performed using R package edgeR [25]. The *P*-value significance threshold in multiple tests was set by the false discovery rate (FDR) [26]. The fold-changes were estimated according to the fragments per kilobase of transcript sequence per million base pairs sequenced (FPKM) in each sample [27]. A cutoff value of 1 FPKM was used as the detection limit. Differentially expressed RNAs with fold change > |2| and *q*-value (FDR adjusted *P*-value) < 0.05, considered significantly modulated, were retained for further analysis. The RNA-seq data have been deposited in Gene Expression Omnibus (GEO) under the accession number GSE177029.

Transcriptomic differences of placentas have been reported in a large cohort of subjects with 94 PE, 56 FGR, and 155 control placental samples using RNA-seq (European genome-phenome archive, https://www.ebi.ac.uk/ega, accession codes: EGAD00001003457, EGAD00001003507, EGAD00001003508, EGAD00001006304, and EGAD00001004860) [28]. We compared the gene expression profiles of normal control placental samples from the current study and the above report. The correlation of FPKM values of the two groups was analyzed. Meanwhile, transcriptomic changes of SLE placentas were also compared with those from the PE and FGR. Principal component analysis [29] was used to predict disparities in expression patterns among SLE-, PE-, and FGR-DEGs.

### Functional genomic analysis of dysregulated genes

To explore biological functions and involved signaling pathways of DEGs, the Gene Ontology (GO) and Kyoto Encyclopedia of Genes and Genomes (KEGG) enrichment analyses of the DEGs were conducted using the Metascape analysis tool [30]. A minimum count of 3 was considered positive expression. Genes with enrichment factor > 1.5 and *P* < 0.05 were considered statistically significant. The GO and KEGG terms fulfilling this condition were defined as significantly enriched GO and KEGG terms.

CIBERSORTx analysis was conducted to estimate the fraction of twenty-two types of immune cells in placental tissues [31]. The non-parametric test (Mann-Whitney *U* test) was performed to illuminate the different cell components between SLE and normal term placental samples.

### Gene co-expression network analysis

To study relationships between dysregulated lncRNAs and mRNAs in SLE placentas, we calculated co-expression relationships between lncRNAs and mRNAs according to the dynamic change of gene expression signal value and obtained the expression regulation relationship and direction between genes to construct the gene expression regulation network. Using the co-expression network, we can analyze the gene regulation ability and obtain the core regulatory genes. The co-expression network was constructed using Cytoscape [32]. Pearson correlation coefficients were used for the lncRNAs-mRNAs co-expression network when they were above 0.90.

### Cis- and trans-target gene prediction of lncRNAs

RNAplex was used to identify potential targets of differentially expressed lncRNAs. The lncRNA *cis*-action is predicted by searching all coding genes within the 10-kb upstream and downstream of the target lncRNA, and these neighboring genes may be regulated by lncRNAs. The lncRNA *trans*-action was predicted based on nucleic acid base pairing [33]. The GO and KEGG enrichment analyses of the lncRNA target genes were conducted.

### Real-time quantitative PCR (RT-qPCR)

To validate the RNA-seq data, six lncRNAs with different expression patterns were selected for RT-qPCR [34] using NuHi Robustic SYBR Green Mix. A new set of samples (*n* = 8/group with four female and four male fetuses; Additional file 2: Table S2) was used, in which six samples overlapped with ones used in RNA-seq analysis. Data were normalized to GAPDH. Primers are listed in Additional file 3: Table S3. The normalized data were analyzed using the 2^−ΔΔCT^ method [34].

### Statistical analyses

Data were expressed as means ± standard deviation or medians with range. Comparison of continuous data was performed by independent Student’s *t* test. Microsoft Excel (2016) and SigmaPlot (13.0) for Windows were used for statistical analyses. *P*-values < 0.05 were considered statistically significant.

## Results

### Patient characteristics

Demographic and clinical characteristics are shown in Table 1 and Additional file 1: Table S1. All SLE patients were on maintenance medication (prednisone ≤ 15 mg daily and hydroxychloroquine 200–400 mg daily). Maternal age and body mass index (BMI) were similar between SLE and NT. The mean newborn body weight and gestation age in SLE were significantly (*P* < 0.05) lower than those in NT. Eight patients, each in NT and SLE groups, underwent vaginal delivery.

### RNA-seq analysis

Deep sequencing generated the mean number of raw reads, 67,745,864 and 66,918,866, for SLE and NT groups, respectively. Removing reads with non-canonical letters or with low quality and discarding the sequences shorter than 25 nucleotides, the mean numbers of clean reads were 66,385,880 and 65,846,828 for SLE and NT groups, respectively, which were retained for analysis.

### SLE-dysregulated lncRNAs in placentas

**Fig. 1:**
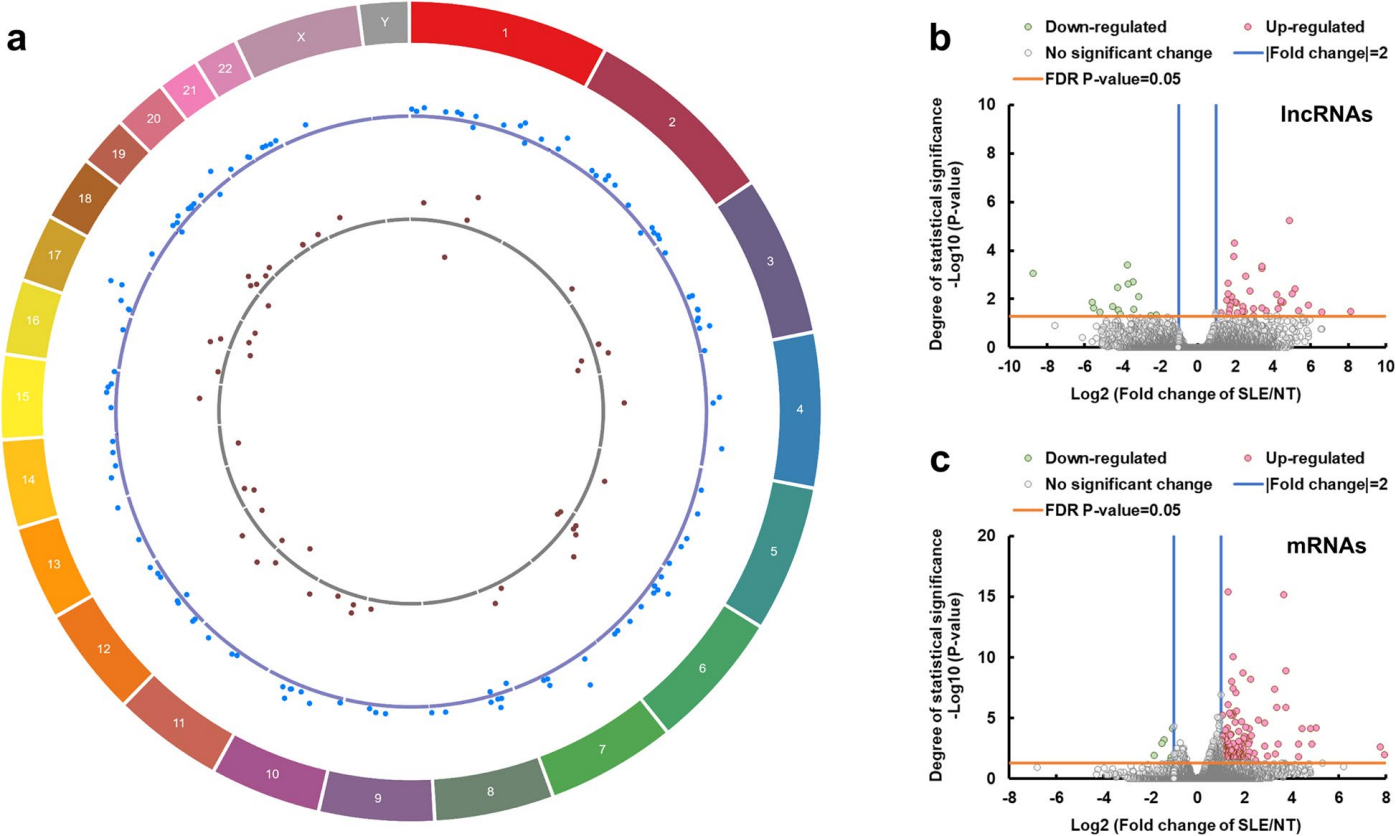
SLE-dysregulated transcriptomic profiles of placentas. **a** Circos plot illustrating the chromosomal position of dysregulated lncRNAs (gray dots) and mRNAs (green dots) between SLE vs. NT. Each dot represents one gene. The numbers and letters in the outer ring indicate the chromosomal location. For each scatter plot track, dots outside and inside of the centerline are upregulated and downregulated genes, respectively. Circos plot was created using Circa program for Windows (OMGenomics). **b** lncRNA volcano plot of SLE vs. NT. **c** mRNA volcano plots of SLE vs. NT. *n* = 8/group

Fifty-two dysregulated lncRNAs were identified (Fig. 1a, b; Additional file 4: Table S4). Of these lncRNAs, 37 were upregulated, and 15 were downregulated. Among these dysregulated lncRNAs, NONHSAT152368.1 (280-fold) was the most upregulated lncRNA, whereas ENST00000602755 (0.0023-fold) was the most downregulated lncRNA. One dysregulated lncRNA (NONHSAT222664.1) was located on X-chromosome and was upregulated. None of these dysregulated lncRNAs was located on Y-chromosome.

### SLE-dysregulated mRNAs in placentas

We also identified 130 dysregulated mRNAs (Fig. 1a, c; Additional file 5: Table S5). Of these dysregulated mRNAs, 122 were upregulated, and 8 were downregulated. *KRT24* (246-fold) was the most upregulated mRNA, and *ANGPT2* (0.28-fold) was the most downregulated mRNA. One dysregulated mRNA (*MAOB*) was located on X-chromosome and was upregulated. None of these dysregulated mRNAs was located on Y-chromosome.

### RNA-seq data comparisons

After trimming off genes with the FPKM <1, we observed that 9780 (93.3%) protein-coding genes identified in our normal term cohorts (Additional file 6: Table S6) were identical to those reported in Gong’s study [28]. Analysis of FPKM values showed a significant (*r* = 0.473, *P* <0.001, Additional file 7: Fig. S1) correlation between these two groups, suggesting similar expression profiles between these two different cohorts. In addition, 17 DEGs were overlapped in SLE (Additional file 8: Fig. S2) and PE placentas, in which 11 were upregulated in SLE but downregulated in PE placentas and one downregulated in SLE but upregulated in PE placentas. Likewise, four DEGs were overlapped between SLE- and FGR-placentas, in which one was upregulated in SLE but downregulated in FGR placentas. Principal component analysis showed disparities in expression patterns among SLE-, PE-, and FGR-DEGs (Additional file 9: Fig. S3). No overlap was identified among SLE-, PE-, and FGR-dysregulated lncRNAs.

### Functional genomics analysis of dysregulated genes

**Fig. 2:**
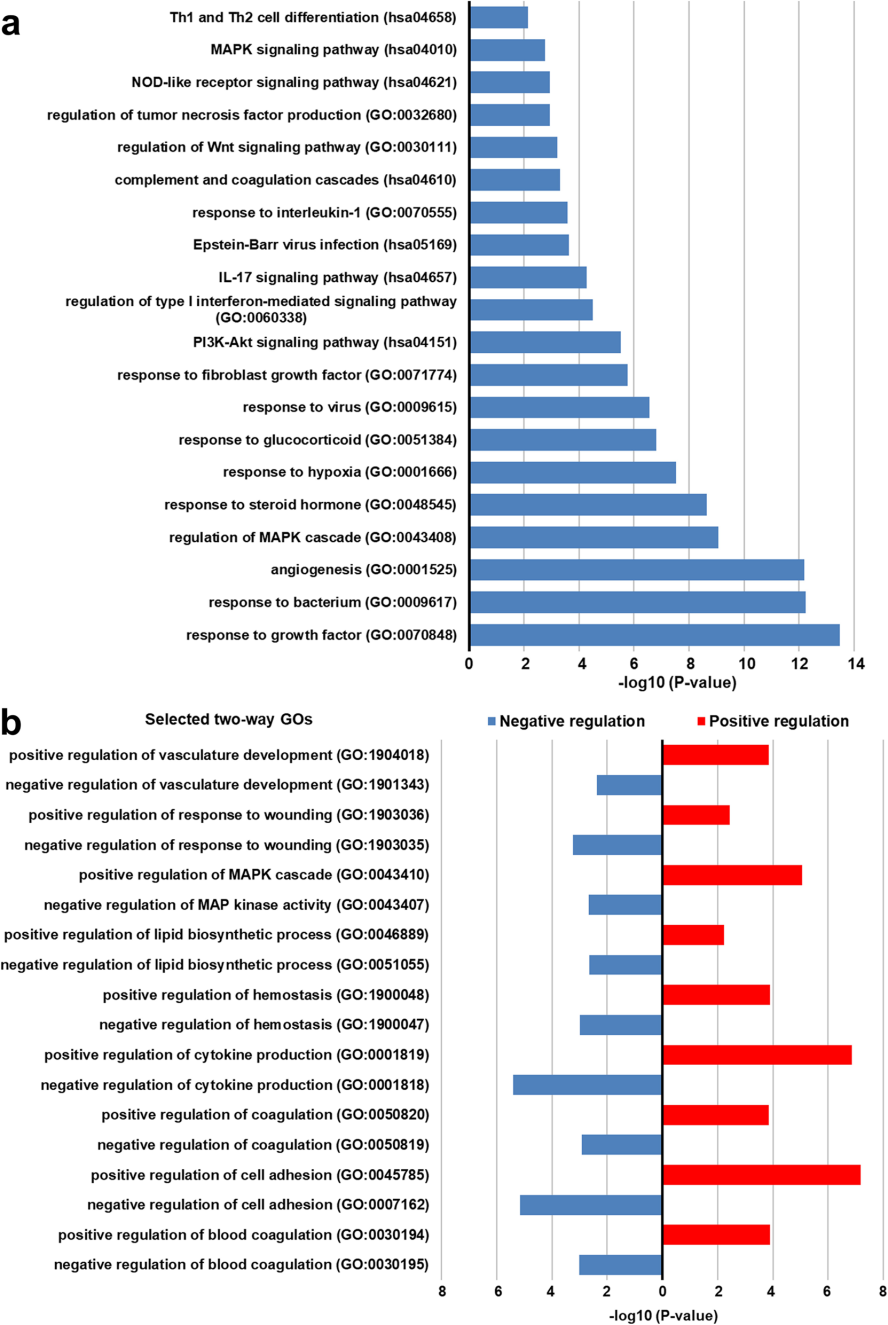
Enrichment analysis for SLE-dysregulated mRNAs in placentas. **a** Selected GOs and KEGGs in SLE. **b** Selected two-way enrichment

We performed bioinformatics analysis with 130 SLE-dysregulated mRNAs, aiming to explore their biological functions and gene networks in SLE placentas. We found that SLE-dysregulated genes were highly enriched in signaling pathways, including regulation of type I interferon-mediated signaling pathway, response to hypoxia, regulation of MAPK (mitogen-activated protein kinase) cascade, response to steroid hormone, complement and coagulation cascades, and Th1 and Th2 cell differentiation (Fig. 2a). In addition, positive or negative regulation of biological functions, such as regulation of vasculature development, cell adhesion, blood coagulation, hemostasis, and response to wounding, was also enriched (Fig. 2b). These findings suggest that a variety of placental function-associated genes/pathways were involved in the pathogenesis of SLE pregnancy.

**Fig. 3:**
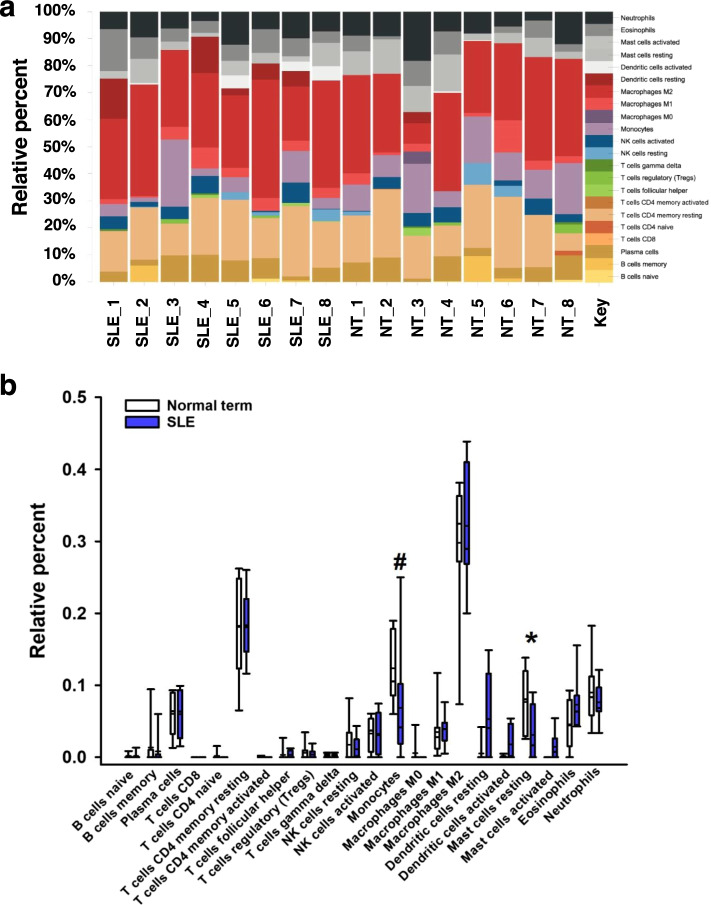
CIBERSORTx algorithm analysis. **a** Bar chart showing the percentage of 22 kinds of immune cells in each sample by estimating relative subsets of RNA transcripts. **b** Box plot showing the fraction of 22 immune cells in NT and SLE placentas by Mann-Whitney *U* test. * *P* < 0.05, **#** *P* = 0.05

CIBERSORTx algorithm was used to estimate the fraction of twenty-two types of immune cells in placentas (Fig. 3 and Additional file 10: Table S7). Macrophages M2 (alternative activated macrophages), T cell CD4 memory resting, monocytes, and neutrophils were top enriched immune cells (Fig. 3a and Additional file 10: Table S7). The results of the Mann-Whitney *U* test suggested that monocytes were differentially enriched between SLE and NT placentas (Fig. 3b).

### Co-expression network of lncRNAs and mRNAs

A co-expression network was established to determine the interactions of dysregulated lncRNAs and mRNAs (Additional file 11: Fig. S4). We found a co-expression network composed of 14 lncRNAs and 39 mRNAs. This network showed that one lncRNA (NONHSAT192274.1) could target at least 16 mRNAs, and one mRNA (*FN1*) could correlate with at least six lncRNAs. NONHSAT192274.1 and NONHSAT112918.2 were the most connected lncRNAs. *VEGFA* was one of the most connected mRNAs. These results strongly support a network in which lncRNAs and mRNAs function together in placentas during SLE pregnancy.

### LncRNA target prediction analysis

The “*cis*” and “*trans*” analyses are shown in Additional file 12: Table S8 and Additional file 13: Table S9, respectively. The prediction results indicated that 49 of 52 SLE-dysregulated lncRNAs may target 1429 genes. The two remaining lncRNAs had no potential target genes.

**Fig. 4:**
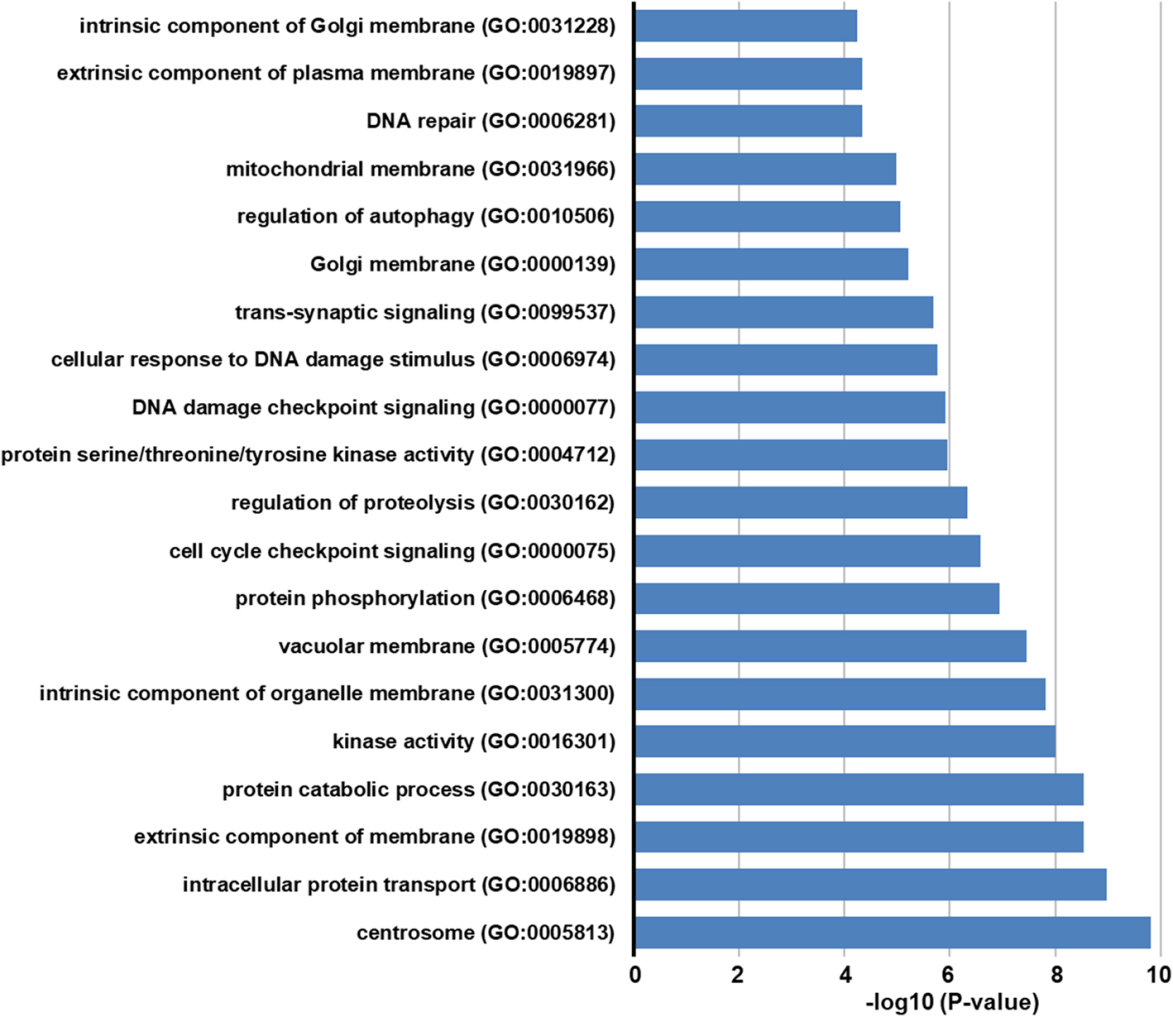
Enrichment analysis for lncRNA target genes. SLE-dysregulated lncRNAs may target 2387 genes including both “*cis*” and “*trans*” genes. Biological functions and involved signaling pathways of these 2387 genes were explored using Metascape online analysis tool

Bioinformatics analysis was conducted to predict the biological roles of target genes of SLE-dysregulated lncRNAs (Fig. 4). The GO project enrichment of target genes included 165 biological processes, 39 cellular components, and seven molecular functions (*P* < 0.05), mainly involving intracellular protein transport, protein catabolic process, kinase activity, DNA damage checkpoint signaling, and mitochondrial membrane. Hence, lncRNAs may affect these biological processes, cellular components, and functions, contributing to the pathogenesis of SLE.

### Confirmation of dysregulated lncRNAs

**Fig. 5:**
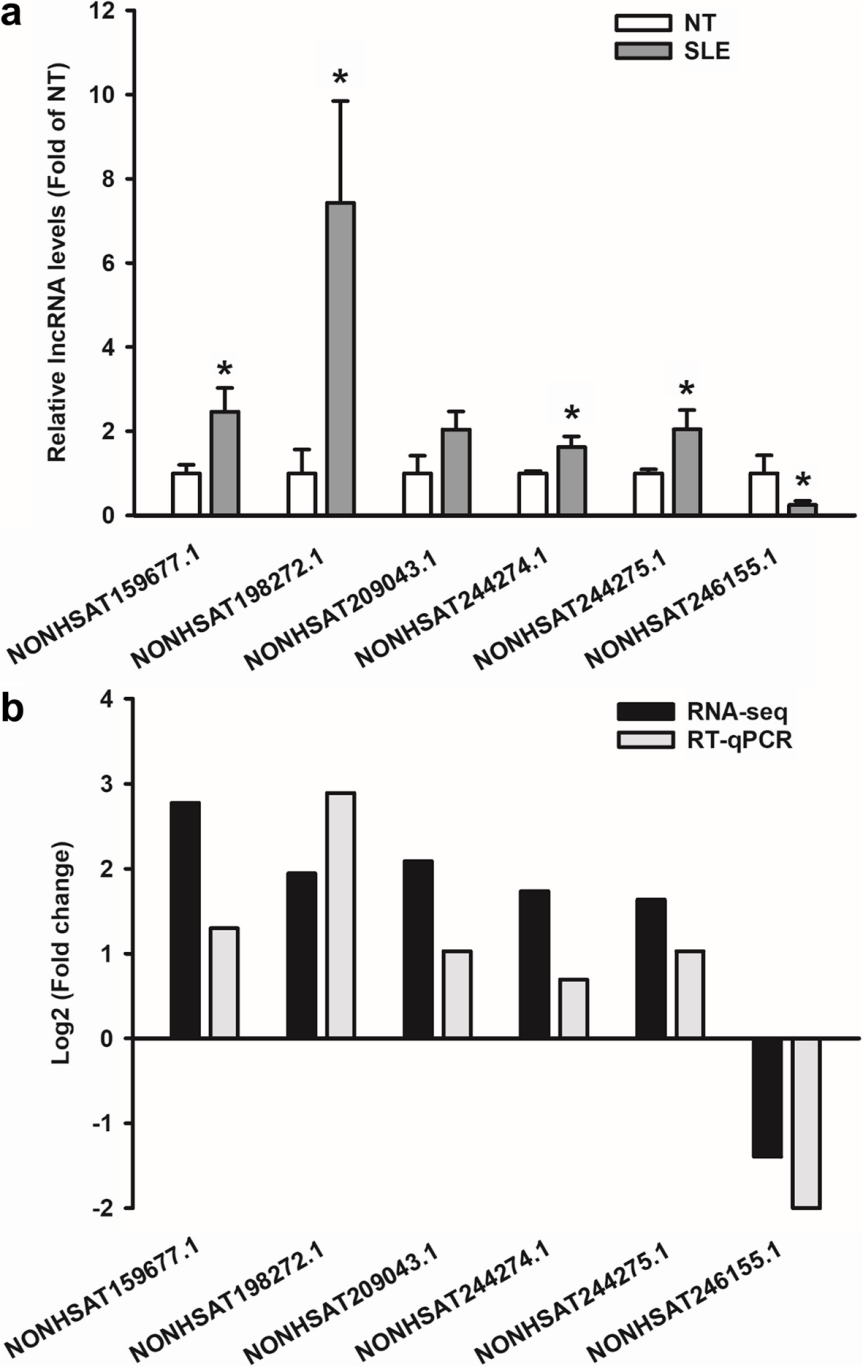
Validation of SLE-dysregulated lncRNAs by RT-qPCR. **a** Relative lncRNA levels (fold of NT control) in SLE. **b** The change patterns between RNA-seq and RT-qPCR. *Differ (*P* < 0.05) from NT. *n* = 8/group

Consistent with RNA-seq data (Fig. 5a), NONHSAT159677.1, NONHSAT198272.1, NONHSAT244274.1, and NONHSAT244275.1 were upregulated, and NONHSAT246155.1 was downregulated (Fig. 5b). The level of NONHSAT209043.1 exhibited a similar upregulation trend as seen in the RNA-seq analysis, but this upregulation did not reach significance (Fig. 5).

## Discussion

In this study, we defined, for the first time, the lncRNA expression profiles of human placentas from SLE pregnancies, though lncRNA expression profiles of human SLE have been reported in peripheral blood mononuclear cells (PBMCs) [17, 18, 20–22, 35, 36], monocyte-derived dendritic cells [37], and whole blood [38]. Our RNA-seq analysis revealed 52 dysregulated lncRNAs in SLE placentas with more upregulated genes than downregulated, which agrees with the pattern in monocyte-derived dendritic cells [37] but differs from the patterns reported in PBMCs and whole blood from SLE patients [17, 38]. Among these dysregulated lncRNAs, NONHSAT192272.1 and NONHSAT192274.1 have been reported in whole blood [38], while NONHSAT022132.2 has been found in PBMCs from SLE patients [36]. Interestingly, all the above three genes in SLE placentas show different expression patterns compared with blood samples. For instance, NONHSAT192272.1 and NONHSAT192274.1 are upregulated in SLE placentas whereas downregulated in SLE whole blood. NONHSAT022132.2 is downregulated in SLE placentas but upregulated in SLE PBMCs. This differential dysregulation of lncRNAs might be due to tissue-specific expression of lncRNAs in human tissues [39]. Specifically, the placenta comprises different cell types, including syncytiotrophoblasts, cytotrophoblasts, mesenchymal cells, mesenchymal-derived macrophages, fibroblasts, vascular smooth muscle cells, perivascular cells, endothelial cells, and blood cells in the intervillous space and fetal vessels [40, 41]. The diverse cell types in the placenta tissue may also contribute to the above different profiles.

CIBERSORTx analysis predicted an enrichment of monocytes in SLE placentas, suggesting that SLE robustly dysregulated RNA transcripts in placental monocytes. However, CIBERSORTx, and other analytical tools, such as xCell, TIMER, and EPIC, may not yet be able to predict gene expression profiles of trophoblasts and other placental cells [42], single-cell RNA-seq may do so. SLE and PE pregnancies have similar patterns in placental protein expressions [43]. In this study, we compared our RNA-seq data with the other dataset from a large cohort of subjects with 94 PE, 56 FGR, and 155 control placentas [28]. However, only a small portion of genes are commonly dysregulated (Additional file 8: Fig. S2). Further principal component analysis indicates huge disparities in expression patterns among SLE-, PE-, and FGR-DEGs. Interestingly, one gene (Follistatin Like 3, *FSTL3*) was upregulated in placentas from SLE, PE, and FGR. FSTL3 is a regulatory glycoprotein that is upregulated in PE and FGR maternal serum and in PE placentas [28, 44]. Hypoxia, which is a biological function enriched in SLE placentas (Fig. 2a), increased the protein levels of FSTL3 in human trophoblasts [45]. In addition, knockdown of *FSTL3* suppressed proliferation and migration of human trophoblasts in vitro [45]. Similar to PE and FGR, which are characterized by maternal systemic inflammation [46, 47], SLE is defined as an autoimmune disease with acute and chronic inflammation [48]. Thus, this commonly upregulated gene in placentas from SLE, PE, and FGR may be important in controlling inflammation responses in placentas.

The distribution of SLE-dysregulated mRNAs and lncRNAs on different chromosomes was also observed. One SLE-dysregulated mRNA and one lncRNA are located on X-chromosome, while none is on Y-chromosome, suggesting that sex chromosome may be minimally involved in sex-dimorphic regulation of gene expression in SLE placentas. The X chromosome contains a large number of genes that are partly responsible for the hyperresponsiveness of the female immune system [49], and the Y chromosome contributes to autoimmunity in mouse models of SLE [50, 51].

Our bioinformatics analysis revealed intriguing biological functions of these dysregulated protein-coding genes in SLE placentas. In particular, both positive and negative regulation of biological processes, such as positive regulation of cell adhesion and negative regulation of cell adhesion were enriched (Fig. 2b). The above two-way regulations indicate that SLE placentas are undergoing comprehensive regulations during pregnancy.

It remains to be elusive what causes these dysregulations of lncRNAs. One major factor might be tissue oxygen levels, as the response to hypoxia is recognized as an SLE-dysregulated network (Fig. 2a). This is supported by the current observation that higher expression of VEGFA, which is hypoxia-driven [52]. Enrichment of response to hypoxia in SLE-induced differential expression lncRNAs suggests that placentas from SLE pregnancies may undergo hypoxia compared with controls. Thus, as hypoxia (~1.5% O_2_) inhibits extravillous trophoblasts outgrowth and proliferation [53], SLE-associated hypoxia may decrease placental weight, leading to FGR [4–6]. This is supported by our current finding that the mean newborn body weight from SLE pregnancies is significantly lower than controls.

SLE-dysregulated genes in placentas were also enriched in type I interferon-mediated signaling pathway, which plays a pivotal role in the pathogenesis of SLE [54]. Genome-wide association studies (GWAS) of SLE patients have shown that key genetic variants are involved in over-activation or regulatory deficits in the innate immune responses that are closely correlated to type I interferons (IFNs) [55]. Additionally, risk alleles that operate in IFN pathway genes have also been implicated in the pathogenesis of lupus in GWAS [56]. The placenta is a transient organ. However, its involvement with the type I interferon signaling pathway suggests that the placenta is indeed a target of SLE during pregnancy, which may cause placental dysfunctions, contributing to maternal and fetal complications commonly seen during SLE pregnancy.

Our findings from the lncRNA-mRNA co-expression and lncRNA target prediction strongly support that lncRNAs and mRNAs interact and function together in placentas during SLE pregnancy. This is in line with the reports that similar lncRNA-mRNA co-expression networks in SLE monocyte-derived dendritic cells and PBMCs [17, 37]. Moreover, *VEGFA*, one of the potent regulators for endothelial growth and function [57], was identified as one of the most connected mRNAs. Given that dysregulated mRNAs in SLE placentas were also enriched in the function of angiogenesis (Fig. 2a), our data suggest that the lncRNA-mRNA co-expression network may be actively involved in the dysregulation of vascular functions during SLE pregnancies.

Dysregulation of lncRNA has been reported to be associated with the severity of SLE. For example, lncRNA NEAT1 levels are significantly higher in PBMCs of SLE patients compared with the healthy group and are positively correlated with SLE disease activity [20]. GAS5 is downregulated in PBMCs and plasma from SLE patients and is negatively correlated with SLE disease activity [21, 58]. Linc0597 and linc0949 are downregulated in PBMCs from SLE patients and negatively correlated with SLE disease activity [21]. In the current study, these lncRNAs (NEAT1, GAS5, linc0597, and linc0949) were not significantly dysregulated in SLE placentas, indicating different lncRNA expression profiles in different SLE tissues. Searching and validating biomarkers for SLE during pregnancy requires a combination of clinical manifestations and potential biomarkers. In the present study, NONHSAT159677.1, NONHSAT198272.1, NONHSAT244274.1, NONHSAT244275.1, and NONHSAT246155.1 were demonstrated to be dysregulated in SLE placentas. Expression profiles of NONHSAT198272.1 and NONHSAT246155.1 in human tissues were reported in the lncRNAs database (http://www.noncode.org/). NONHSAT198272.1 has been observed to be expressed in kidney and placenta tissues, whereas NONHSAT246155.1 was reported to be expressed in various human tissues, and the expression level of NONHSAT246155.1 is the second-highest among these tissues. This is the first report that NONHSAT159677.1, NONHSAT244274.1, and NONHSAT244275.1 are expressed in human placentas. Based on the above observations, we speculate that the above SLE-dysregulated lncRNAs may potentially serve as biomarkers for SLE. However, the functions of the above SLE-dysregulated lncRNAs require further study.

In the current study, we define, for the first time, the transcriptomic profiles in placentas from SLE pregnancies. Our findings support the concept that lncRNAs play comprehensive roles in the pathogenesis of SLE placentas. However, the limitations of our study should be acknowledged. First, this study is limited by the small patient number, and further validation of these dysregulated mRNAs and lncRNAs in a larger cohort of patients is needed to confirm our results. Second, the narrow SLEDAI score range (0–6) of SLE patients recruited strongly limits the analysis of the correlation between DEGs expression levels and disease activity. In addition, further functional studies are needed to define the role of these dysregulated lncRNAs in SLE placentas.

## Conclusions

This study demonstrates a comprehensive expression profile of lncRNAs and mRNAs in SLE placentas. The findings suggest regulatory functions of lncRNAs and mRNAs, which are implicated in the development and pathogenesis of SLE pregnancy. SLE-dysregulated lncRNAs may potentially serve as biomarkers for SLE.

## Supplementary Information

**Additional file 1: Table S1.** Clinical manifestations and laboratory records.

**Additional file 2: Table S2.** Patient demographics for RNA-seq and RT-qPCR.

**Additional file 3: Table S3.** RT-qPCR primers.

**Additional file 4: Table S4.** SLE dysregulated lncRNAs.

**Additional file 5: Table S5.** SLE dysregulated mRNAs.

**Additional file 6: Table S6.** Gene expression levels of controls from two separate cohorts.

**Additional file 7: Figure S1.** Correlation analysis of FPKM values of two groups.

**Additional file 8: Figure S2.** Venn diagram showing the overlap between SLE-, PE-, and FGR-dysregulated protein-coding genes.

**Additional file 9: Figure S3.** Principal component analysis of SLE-, PE-, and FGR- dysregulated protein-coding genes.

**Additional file 10: Table S7.** CIBERSORTx analysis.

**Additional file 11: Figure S4.** Co-expression network with dysregulated lncRNAs and mRNAs.

**Additional file 12: Table S8.** The predicted cis-associated genes of the SLE-dysregulated lncRNAs.

**Additional file 13: Table S9.** The predicted trans-associated genes of the SLE-dysregulated lncRNAs.

## Data Availability

The RNA-seq data have been deposited in Gene Expression Omnibus (GEO) under the accession number GSE177029. The datasets used and/or analyzed during the current study are available from the corresponding author on reasonable request.

## Abbreviations

SLE: Systemic lupus erythematosus
FGR: Fetal growth restriction
lncRNAs: Long noncoding RNAs
NF-κB: Nuclear factor-κB
MAPK: Mitogen-activated protein kinase
TNF: Tumor necrosis factor
RNA-seq: RNA sequencing
NT: Normal full-term
SLEDAI: SLE disease activity index
RIN: RNA integrity number
DEG: Differential expression gene
GO: Gene Ontology
KEGG: Kyoto Encyclopedia of Genes and Genomes
FDR: False discovery rate
FPKM: Fragments Per Kilobase of transcript sequence per Millions base pairs sequenced
RT-qPCR: Real-time quantitative PCR
PBMCs: Peripheral blood mononuclear cells
GWAS: Genome-wide association studies
IFNs: Interferons
