# Supplementary material for: Systemic lupus erythematosus dysregulates the expression of long noncoding RNAs in placentas
Source: Arthritis Res Ther. 2022 Jun 14;24:142. doi: 10.1186/s13075-022-02825-7 (PMC9195362; doi:10.1186/s13075-022-02825-7)
Supplement: Supplementary file 2 — Additional file 2: Table S2. Patient demographics for RNA-seq and RT-qPCR. [file 13075_2022_2825_MOESM2_ESM.docx]

| **Table S2** Patient demographics for RNA-seq and RT-qPCR | | | | | | |
| --- | --- | --- | --- | --- | --- | --- |
|  | Diagnosis | N | Maternal BMI | Maternal age (years) | Gestational age (weeks) | Fetal weight (gram) |
| RNA-seq | NT | 8 | 27.24 ± 1.45 | 30.50 ± 0.60 | 39.29 ± 0.24 | 3378.13 ± 102.56 |
|  | SLE | 8 | 26.43 ± 1.23 | 29.75 ± 1.16 | 37.98 ± 0.56 | 2956.25 ± 196.50 |
|  | *P*-value | | > 0.05 | > 0.05 | < 0.05 | < 0.05 |
| RT-qPCR | NT | 8 | 27.14 ± 1.37 | 31.13 ± 0.61 | 39.04 ± 0.18 | 3366.25 ± 87.51 |
|  | SLE | 8 | 26.92 ± 1.35 | 30.88 ± 1.25 | 37.86 ± 0.52 | 2906.25 ± 136.75 |
|  | *P*-value | | > 0.05 | > 0.05 | < 0.05 | < 0.05 |
| ***RNA-seq* RNA sequencing, *RT-qPCR* Real time quantitative PCR, *BMI* Body mass index*, NT* normal term*, SLE* Systemic lupus erythematosus** | | | | | | |
