# Supplementary material for: Systemic lupus erythematosus dysregulates the expression of long noncoding RNAs in placentas
Source: Arthritis Res Ther. 2022 Jun 14;24:142. doi: 10.1186/s13075-022-02825-7 (PMC9195362; doi:10.1186/s13075-022-02825-7)
Supplement: Supplementary file 3 — Additional file 3: Table S3. RT-qPCR primers. [file 13075_2022_2825_MOESM3_ESM.docx]

| **Table S3** RT-qPCR primers | | |
| --- | --- | --- |
| Gene name | Forward (5’-3’) | Reverse (5’-3’) |
| NONHSAT159677.1 | TGGACACTGGGCAGAGAAAT | GGTGGGATGGAGAAGACAAA |
| NONHSAT198272.1 | CGCCCCACATCTCTAAACTC | CCCCTCCAGCTTTCATCTTC |
| NONHSAT209043.1 | ATGCTTATCTGCCATGTCCA | GAACTGTCCAAAGGCGGAAA |
| NONHSAT244274.1 | GAGCAGTCCCCTTTGGAGA | CAGGCAGATAACGCACAGG |
| NONHSAT244275.1 | TTTCCACTGCCTTCCACATT | GGATGGGTCAGCTCTTTTTC |
| NONHSAT246155.1 | AATGTTGGTGCTTTTCCTCCT | GAATCGAGGACACACTCACCT |
| GAPDH | TGACTTCAACAGCGACACCCA | CACCCTGTTGCTGTAGCCAAA |
| ***RT-qPCR* Real time quantitative PCR, *GAPDH* glyceraldehyde-3-phosphate-dehydrogenase** | | |
