# Supplementary figures and images for: Systemic lupus erythematosus dysregulates the expression of long noncoding RNAs in placentas
Source: Arthritis Res Ther. 2022 Jun 14;24:142. doi: 10.1186/s13075-022-02825-7 (PMC9195362; doi:10.1186/s13075-022-02825-7)

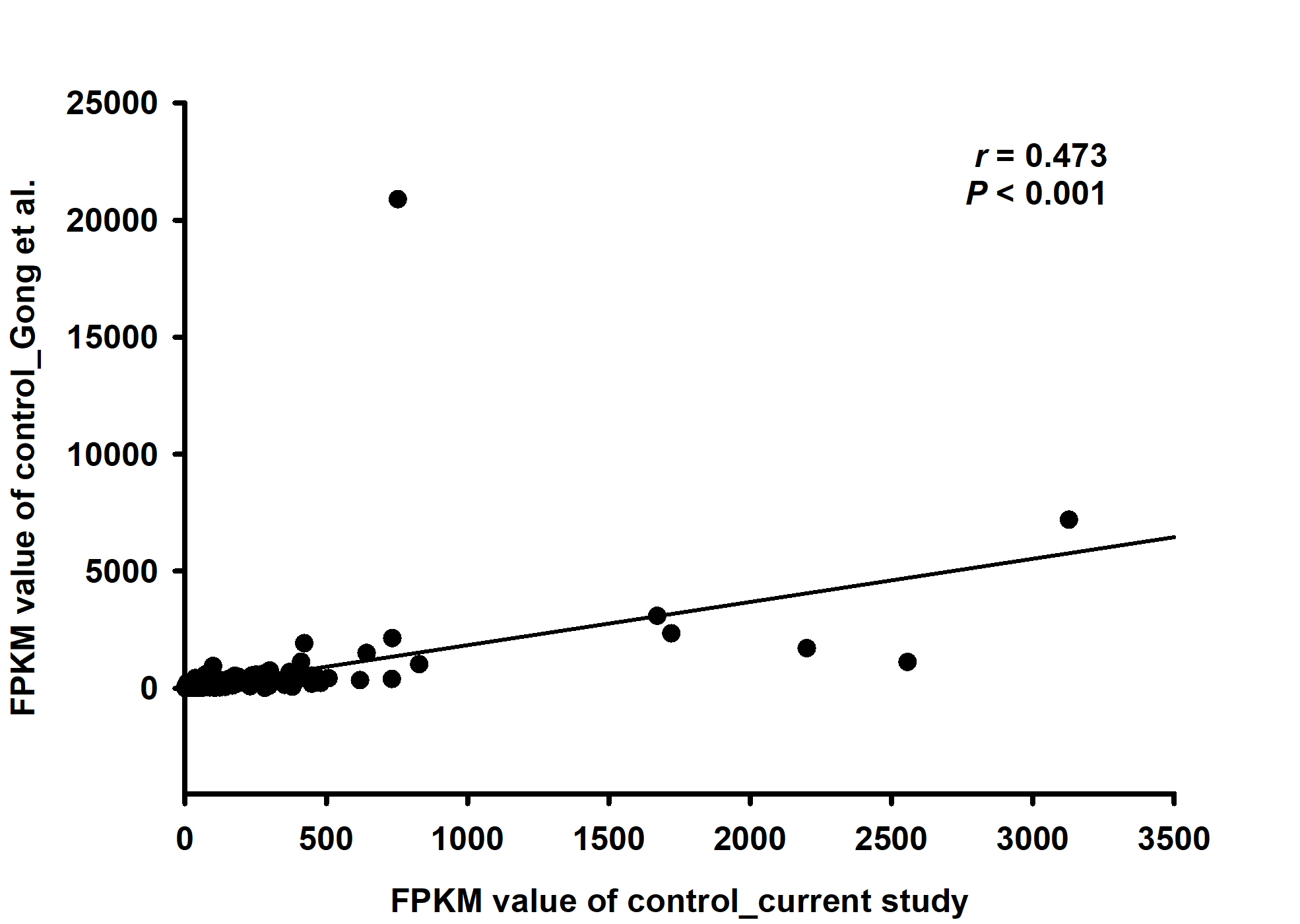

Supplement: Supplementary file 7 — Additional file 7: Figure S1. Correlation analysis of FPKM values of two groups. [file 13075_2022_2825_MOESM7_ESM.jpg]

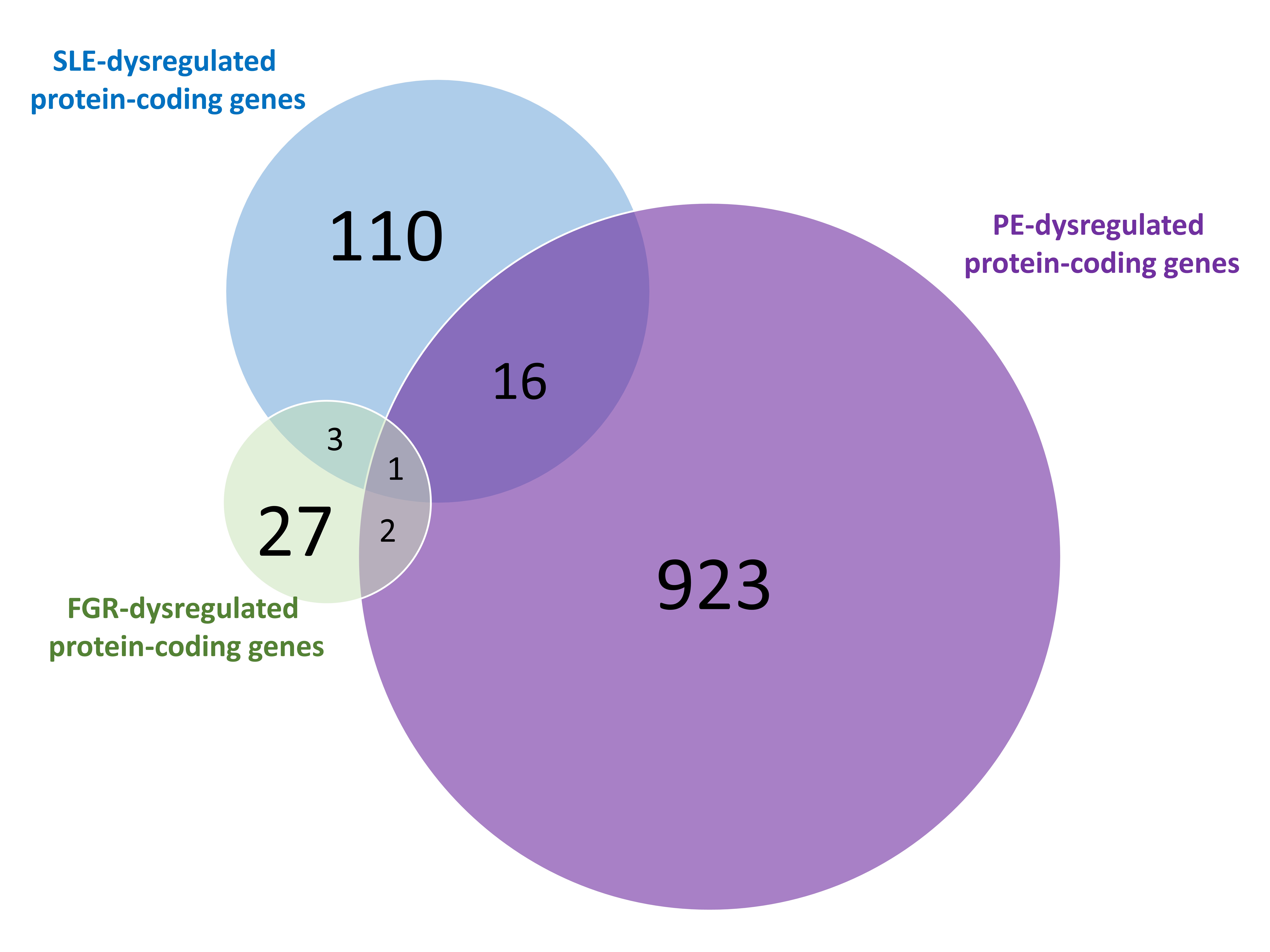

Supplement: Supplementary file 8 — Additional file 8: Figure S2. Venn diagram showing the overlap between SLE-, PE-, and FGR-dysregulated protein-coding genes. [file 13075_2022_2825_MOESM8_ESM.jpg]

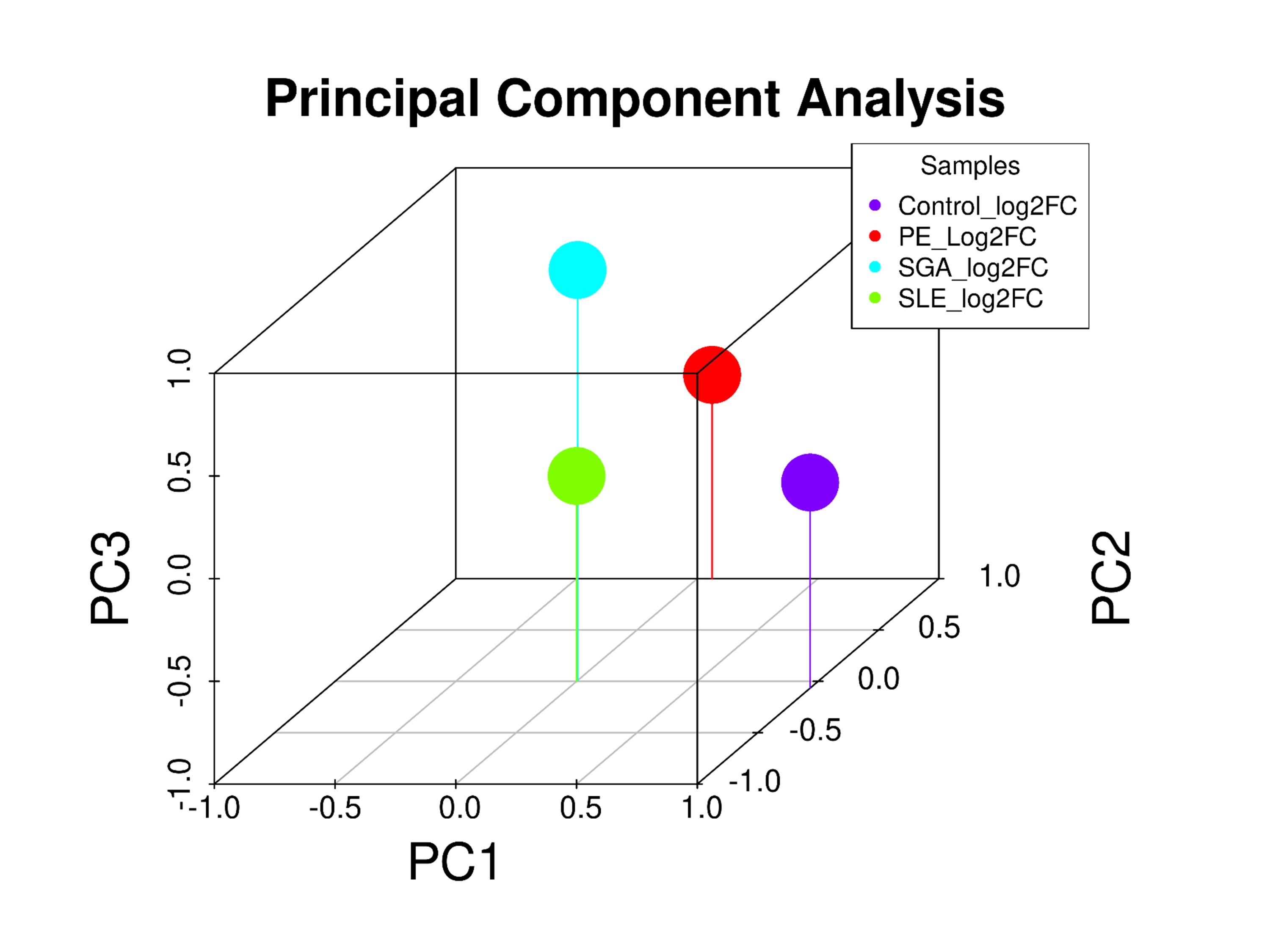

Supplement: Supplementary file 9 — Additional file 9: Figure S3. Principal component analysis of SLE-, PE-, and FGR- dysregulated protein-coding genes. [file 13075_2022_2825_MOESM9_ESM.jpg]

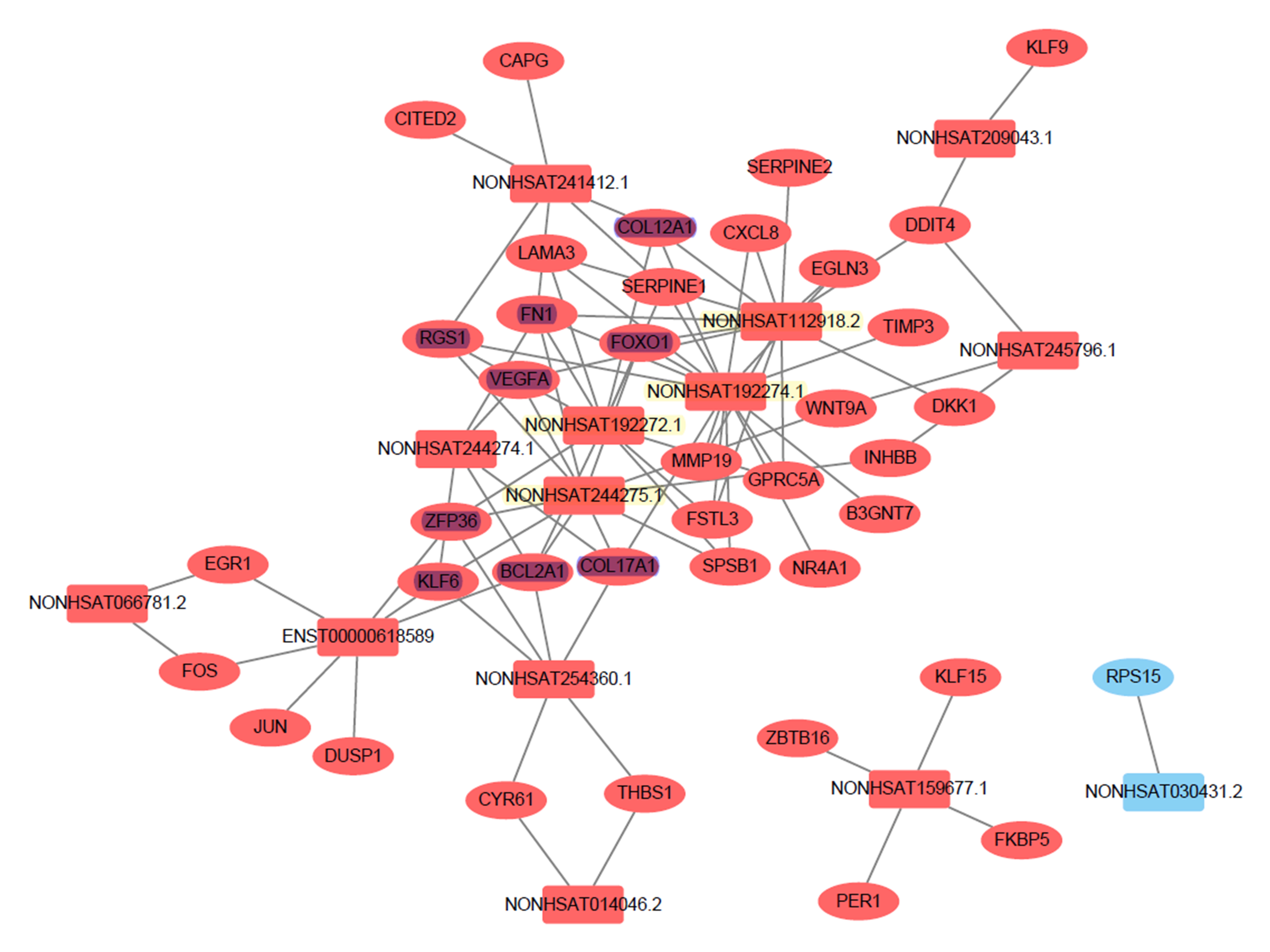

Supplement: Supplementary file 11 — Additional file 11: Figure S4. Co-expression network with dysregulated lncRNAs and mRNAs. [file 13075_2022_2825_MOESM11_ESM.jpg]
